# The Pharmacological and Structural Basis of the AahII–Na_V_1.5 Interaction and Modulation by the Anti-AahII Nb10 Nanobody

**DOI:** 10.3389/fphar.2022.821181

**Published:** 2022-02-28

**Authors:** Riadh Hmaidi, Ayoub Ksouri, Rahma Benabderrazek, Viviane Antonietti, Pascal Sonnet, Mathieu Gautier, Balkiss Bouhaouala-Zahar, Halima Ouadid-Ahidouch

**Affiliations:** ^1^ Laboratory of Biomolecules, Venoms, and Theranostic Applications, Institut Pasteur Tunis, University of Tunis El Manar, Tunis, Tunisia; ^2^ Laboratory of Cellular and Molecular Physiology UR 4667, UFR of Sciences, University of Picardie Jules Verne, Amiens, France; ^3^ Infectious Agents, Resistance and Chemotherapy UR 4294, UFR of Pharmacy, University of Picardie Jules Verne, Amiens, France; ^4^ Medical School of Tunis, University of Tunis El Manar, Tunis, Tunisia

**Keywords:** scorpion toxin, AahII, Na_v_1.5, nanobody, patch-clamp, docking, inactivation

## Abstract

Scorpion α-toxins are neurotoxins that target the fast inactivation mechanism of voltage-gated sodium (Na_V_) channels leading to several neuro- and cardiotoxic effects in mammals. The toxin AahII is the most active α-toxin from the North African scorpion *Androctonus australis Hector* that slows the fast inactivation of Na_V_ channels. To fight scorpion envenomation, an anti-AahII nanobody named NbAahII10 (Nb10) was developed. The efficiency of this nanobody has been evaluated *in vivo* on mice, but its mechanism of action at the cellular level remains unknown. Here we have shown that AahII toxin slows the fast inactivation of the adult cardiac Na_V_1.5 channels, expressed in HEK293 cells, in a dose-dependent manner, while current amplitude was not affected. The inactivation of Na_V_1.5 is slower by a factor of 4, 7, and 35 in the presence of [AahII] at 75, 150, and 300 nM, respectively. The washout partially reversed the toxin effect on inactivation from 8.3 ± 0.9 ms to 5.2 ± 1.2 ms at 75 nM. We have also demonstrated that the highly neutralizing Nb10 can fully reverse the effect of AahII toxin on the channel inactivation kinetics even at the 1:1 M ratio. However, the 1:0.5 M ratio is not able to neutralize completely the AahII effect. Therefore, the application of Nb10 promotes a partial abolishment of AahII action. Bioinformatic analysis and prediction of Na_V_1.5-driven docking with AahII show that Ala39 and Arg62 of AahII play a crucial role to establish a stable interaction through H-bound interactions with Gln1615 and Lys1616 (S3–S4 extracellular loop) and Asp1553 (S1–S2 loop) from the voltage-sensing domain IV (VSD4) of Na_V_1.5, respectively. From this, we notice that AahII shares the same contact surface with Nb10. This strongly suggests that Nb10 dynamically replaces AahII toxin from its binding site on the Na_V_1.5 channel. At the physiopathological level, Nb10 completely neutralized the enhancement of breast cancer cell invasion induced by AahII. In summary, for the first time, we made an electrophysiological and structural characterization of the neutralization potent of Nb10 against the α-scorpion toxin AahII in a cellular model overexpressing Na_V_1.5 channels.

## Introduction

Voltage-gated sodium (Na_V_) channels are large transmembrane proteins responsible for the initiation and propagation of action potentials in excitable cells (Ahern et al., 2016). After voltage-dependent opening, Na_V_ channels undergo a rapid spontaneous inactivation process that terminates Na^+^ permeation (Armstrong et al., 1973; Ahern et al., 2016). Fast inactivation is the main hallmark of eukaryotic Na_V_ channel function that allows cells to repolarize and Na_V_ channels to become ready for reactivation (Ulbricht 2005; Ahern et al., 2016). Eukaryotic Na_V_ channels count 9 isoforms (Na_V_1.1–Na_V_1.9), encoded by 9 distinct genes (SCN1–5A and SCN8–11A) with almost 50% of homology in the amino acid sequences (Goldin et al., 2000; Catterall et al., 2005). These channels consist of the heteromeric assembly of an α-subunit which forms the channel pore and provides its function with two auxiliary β-subunits. The α-subunit of Na_V_ channels contains four homologous, nonidentical domains, each consisting of six transmembrane segments (S1–S6) (Ren et al., 2001; Catterall 2014). Each domain is organized into two parts, the voltage-sensing domain (VSD) from S1 to S4 and the pore module (PM) between S5 and S6 (Catterall et al., 2017). The S4 segments known as voltage sensors modules are positively charged due to four to eight arginine/lysine residues flanked by two hydrophobic residues (Ahern et al., 2016; Catterall et al., 2017). This positively charged motif serves as a gating charge and moves outward upon depolarization to initiate the channel activation.

Rapid inactivation of Na_V_ channels results from the occlusion of the pore by a cytosolic inactivation motif that consists of a cluster of hydrophobic residues, isoleucine, phenylalanine, and methionine (IFM) in the intracellular loop connecting domain III (DIII) and DIV (Armstrong et al., 1973; Goldin 2003). In fact, during the activation process, the movement of the voltage sensors, particularly the DIV voltage sensor (VSD4), exposes a hydrophobic site between the S3 and S4 segments. The binding of the IFM motif to this site leads to a physical blockage of ion movement in the cell (Goldin 2003; Catterall 2014). Because VSD1–3 respond more rapidly to membrane depolarization than VSD4 (Chanda and Bezanilla, 2002), inactivation generally occurs after channel activation. Therefore, the inactivation process is intimately linked to channel activation following the movement of VSD4. Indeed, mutation of the IFM motif can completely abolish fast inactivation in Na_V_ channels (Ahern et al., 2016; Catterall et al., 2017).

Several venomous animals including scorpions have developed an arsenal of toxins that target and disrupt Na_V_ inactivation to immobilize prey or predators (Bosmans and Tytgat, 2007; Hanck and Sheets, 2007). AahII from *Androctonus australis Hector* is the most toxic polypeptide responsible for the noxious effects of the venom with an LD50 < 3 ng at an intracerebroventricular administration in a 20 g mouse (Devaux et al., 2004). At the pharmacological level, AahII is an α-scorpion toxin that targets site 3 on Na_V_ channels and slows the inactivation to sustain sodium influx (Martin et al., 1987; Catterall et al., 2007; Clairfeuille et al., 2019). At the structural level, AahII is a 64-amino-acid peptide stabilized by four disulfide bonds to form a compact β1–α1–β2–β3 scaffold that can highly interact with multiple mammalian Na_V_ channel subtypes (Housset et al., 1994).

To affect the fast inactivation mechanism, AahII interacts with VSD4 by trapping it in a deactivated state. AahII does not disturb channel activation because DI–III voltage sensors can ensure the opening of the channel even if VSD4 remains deactivated. In the absence of AahII, the S4 helix of VSD4 moves outward to unlatch the intracellular fast inactivation gating machinery, as described before (Clairfeuille et al., 2019).

Another well-known α-scorpion toxin with a similar effect is LqhIII from *Leiurus quinquestriatus hebraeus* (Jiang et al., 2021). It was recently reported that LqhIII anchors on top of VSD4 and traps the gating charges of the S4 segment in a unique intermediate-activated state stabilized by four ion pairs. This conformation weakens the binding of the fast inactivation gate and favors the opening of the activation gate (Jiang et al., 2021).

In order to counteract human envenoming caused by scorpion stings, several toxin-specific antivenoms were developed using different approaches. The standard immunotherapy method consists of using purified polyclonal antibody F (ab′)2 fragments prepared from equine hyperimmune sera (Chippaux and Goyffon, 1998; Bouaziz et al., 2008). However, the use of these antibody fragments of ≈100 kDa is only moderately effective due to their polyclonal nature and may cause dangerous adverse effects such as anaphylactic shocks (Pepin-Covatta et al., 1996).

Another method based on the use of murine monoclonal antibodies was later developed to neutralize the effect of the AahII toxin. This study led to the development of the murine 4C1 antibody (Bahraoui et al., 1988), subsequently used to develop an AahII-specific scFv (single-chain variable fragment) (Mousli et al., 1999). Similarly, other studies have allowed the development of an scFv against the AahI that belongs to a distinct antigenic and structural group of *Androctonus australis Hector* scorpion toxins (Devaux et al., 2001). Some years later, a bispecific scFv construct against both AahI and AahII toxins was obtained by engineering techniques able to protect mice against the whole *Androctonus australis Hector* venom (Juste et al., 2007).

However, all these constructs mentioned above have the major problem of synchronization of kinetic diffusion which is due to the huge difference between the molecular weight of antibodies (MW of approx. 150 kDa), F (ab′)2 (MW of approx. 100 kDa), and their target toxins (MW of approx. 7 kDa). Likewise, the ScFv fragment was not lastingly effective due to a VH–VL unstable complex interaction. Moreover, the neutralizing capacity remains moderate, and their use as a human therapeutic might still generate an undesirable human anti-mouse antibody response (HAMA).

More recently, we have developed an antivenom using another type of toxin binders based on the variable domains of the dromedary heavy-chain antibodies (HCAbs, heavy-chain antibody) naturally lacking light chains and CH1 domains of heavy chains, named VHHs. The specified VHH variable domain encodes an antibody fragment, also named Nb (nanobody), that represents the smallest, intact, natural antigen-binding fragment (Hmila et al., 2008), which has been used to develop an anti-*Androctonus australis Hector* venom that recognizes specifically the AahI′ toxin from Aah scorpion venom. This anti-AahI′ can neutralize 3 LD_50_ when tested *in vivo* using s.c. injection in Swiss mice.

Later, by the same approach, an Nb that neutralizes the most toxic compound of Aah venom, AahII toxin, was developed. The so-called Nb10 is an anti-AahII that targets a unique epitope on AahII and neutralizes 7 LD_50_ when tested *in vivo* using Swiss mice (Abderrazek et al., 2009). *In silico* studies demonstrated whether Nb10 binds the active site of AahII toxin (Ksouri et al., 2018).

These two Nbs are characterized by their small size (MW of approx. 15 kDa), good stability, high level of expression in prokaryotic systems, high solubility, and suitable specificity. At the preclinical level, the performance of a bispecific NbF12-10 toxin-specific Nb format (including the anti-AahII Nb10) was demonstrated in an envenoming simulated animal model (Hmila et al., 2010; Hmila et al., 2012).

While Nb10 constitutes the best candidate that neutralizes AahII toxic effects, the precise functional and structural mechanism by which Nb10 neutralizes AahII and interacts with the AahII–Na_V_ channel complex remains unknown. Here, we studied at the electrophysiological and structural levels the interactions of a purified AahII toxin and Nb10 nanobody with the Na_V_1.5 channel stably expressed in a HEK cell model. Our data reveal the mode of action and structural basis of AahII and the α-scorpion toxins in general, on the Na_V_1.5 channel, and proposes Nb10 as a promising antivenom candidate against *Androctonus australis Hector* scorpion stings.

## Results

### Effect of AahII on Na_V_1.5 Channel Activity

**FIGURE 1:**
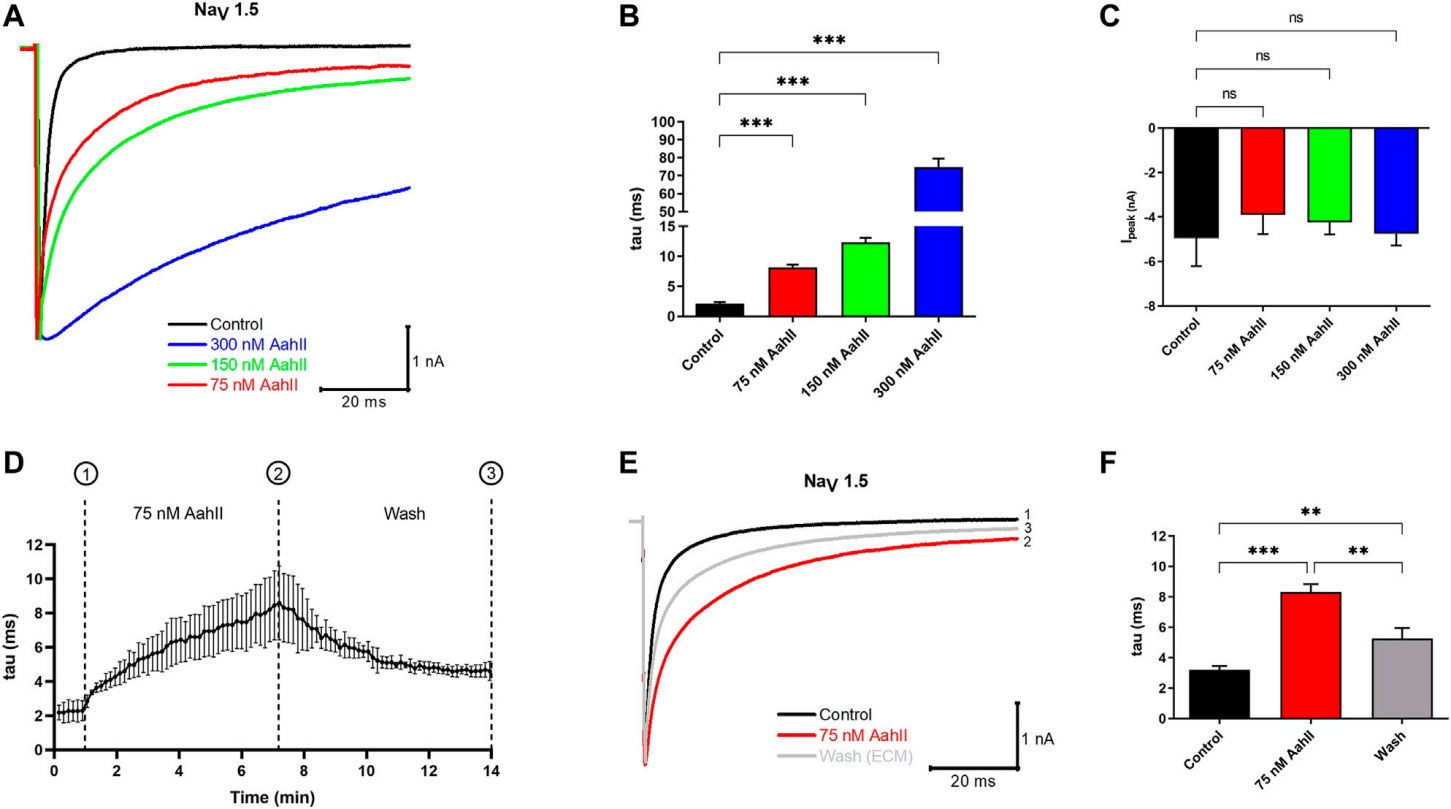
The pharmacological effect of AahII toxin on the Na_V_1.5 channel overexpressed in HEK293 cells. **(A)** Original whole-cell Na^+^ current traces recorded in control condition (control = extracellular medium = ECM) and in the presence of different concentrations of AahII toxin. **(B)** Mean of Na_V_1.5 inactivation time constant (tau) in the control condition and following application of AahII toxin at different concentrations (75, 150, and 300 nM). Values are the mean of 5 independent experiments, *n* = 5. All data are presented as mean ± standard error (***p < 0.001). **(C)** Mean of current amplitude, Ipeak (nA) measured at −40 mV in the same conditions as in panel **(B)**. Values are representative of independent experiments, *n* = 5. All data are presented as mean ± SE (***p < 0.001). **(D)** Mean of the kinetics of inactivation time constant of the Na_V_1.5 channel (tau) following perfusion of AahII toxin at 75 nM from 1 to 7 min followed by washing with ECM up to 14 min. Traces are representative of independent experiments, *n* = 3. All data are presented as mean ± SE. **(E)** Representative of original whole-cell current traces showing the reversible effect of AahII observed in panel **(D)**. Trace 1: current trace recorded in control medium, Trace 2: current obtained at 7 min represents the maximum effect of AahII toxin at 75 nM, and Trace 3: current trace after washing the AahII toxin during 14 min. Traces are representative of independent experiments, *n* = 3. **(F)** Mean of inactivation time constant of the Na_V_1.5 channel (tau) following a similar protocol as in panel **(D)**.

First, we investigated, by patch-clamp technique, the effect of AahII toxin purified from the venom of *Androctonus australis Hector* scorpion on a cardiac sodium channel Na_V_1.5 subunit using the human embryonic kidney cell line HEK293 stably transfected with α subunit of Na_V_1.5 of human origin. The activity of Na_V_1.5 was recorded by a whole-cell patch clamp. In control conditions, Na currents activate rapidly and inactivate within 2–3 ms. Perfusion of increasing concentrations of AahII progressively slows the inactivation kinetics of the Na_V_1.5 channel. This inactivation was slower by a factor of 4, 7, and 35 in the presence of [AahII] at 75, 150, and 300 nM, respectively, when compared to the control conditions (Figures 1A,B; *n* = 5, ***p < 0.001), while current amplitude was not affected (Figure 1C; *n* = 5, ***p < 0.001). In a second series, we investigated whether the effect of AahII was reversible. After current stabilization by perfusing the control solution for 1 min, (AahII) at 75 nM was perfused throughout 7 min until the current was stabilized. Then, the cell was washed by control solution perfusion (Figure 1D; *n* = 3, ***p < 0.001). As expected, AahII slowed the constant time inactivation (tau) from 3.2 ± 0.4 ms in control conditions to 8.3 ± 0.9 ms in AahII treatment conditions at 75 nM (Figures 1E,F; *n* = 3, ***p < 0.001). The washout partially reversed the toxin effect on inactivation to 5.2 ± 1.2 ms (Figures 1E,F; *n* = 3, ***p < 0.001), suggesting its extracellular specific binding.

### The Nb10 Reverses the AahII Effect on Na_V_ Inactivation

**FIGURE 2:**
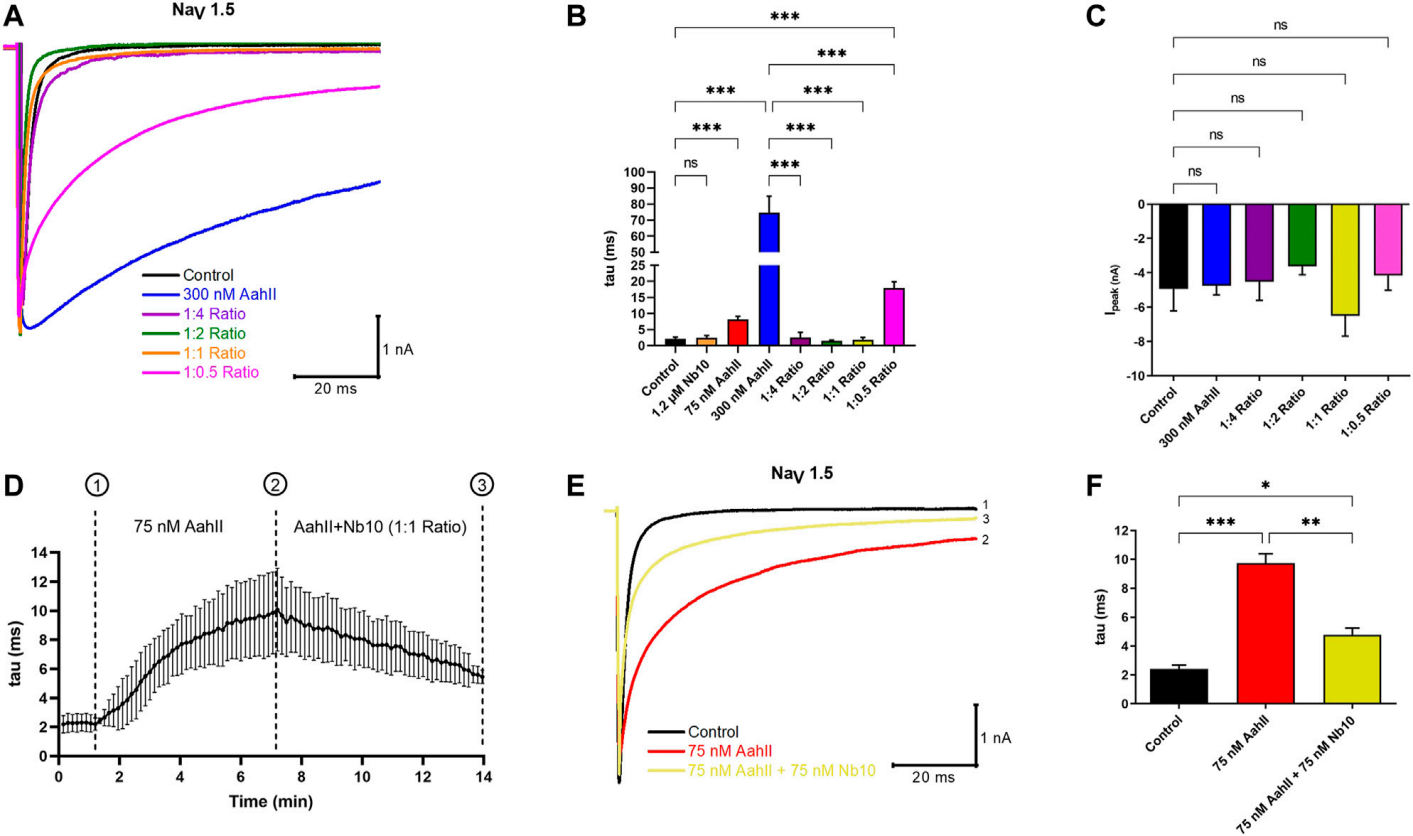
Nb10 interacts with AahII on the Na_V_1.5 channel. **(A)** Original whole-cell Na^+^ current traces recorded at −40 mV in control conditions (Control), in the presence of 300 nM AahII alone, 1.2 µM Nb10 alone, or at different ratios of [AahII]: [Nb10]. 1:4 ratio ([AahII] = 300 nM: [Nb10] = 1.2 µM); 1:2 ratio ([AahII] = 300 nM: [Nb10] = 600 nM), 1:1 ratio ([AahII] = 300 nM: [Nb10] = 300 nM), and 1:0.5 ratio ([AahII] = 300 nM: [Nb10] = 150 nM). **(B)** Mean of Na_V_1.5 channel inactivation time constant (tau) in control conditions (control), AahII, and Nb10 alone and combined at different ratios. Values are representative of independent experiments, *n* = 5. All data are presented as mean ± SE (***p < 0.001). **(C)** Mean of Na^+^ current amplitude recorded at -40 mV in the same conditions as in panel **(A)**. Values are representative of independent experiments, *n* = 5. All data are presented as mean ± SE (***p < 0.001). **(D)** Mean of kinetics of the inactivation time constant of the Na_V_1.5 channel (tau) following perfusion of AahII toxin at 75 nM from 1 to 7 min followed by the perfusion of a mixture of AahII and Nb10 at a 1:1 ratio up to 14 min. Traces are representative of independent experiments, *n* = 3. All data are presented as mean ± SE. **(E)** Representative of whole-cell Na^+^ current traces recorded at -40 mV in the same conditions as in panel **(D)**, Na^+^ current recorded in control conditions (Trace 1), after 7 min of AahII perfusion (Trace 2), and perfusion of AahII and Nb10 at a 1:1 ratio up to 14 min (Trace 3). Traces are representative of independent experiments, *n* = 3. **(F)** Mean of inactivation time constant of the Na_V_1.5 channel (tau) following a similar protocol as in panel (D). Values are representative of independent experiments, *n* = 3. All data are presented as mean ± SE (***p < 0.001).

We investigated whether Nb10 is able to reverse the Na_V_ slow inactivation kinetic induced by 300 nM of AahII by whole-cell patch clamp experiments using four different AahII:Nb10 M ratios. Interestingly, the 1:4 ([AahII] = 300 nM: [Nb10] = 1.2 µM), 1:2 ([AahII] = 300 nM: [Nb10] = 600 nM), and 1:1 ([AahII] = 300 nM: [Nb10] = 300 nM) ratios are able to neutralize the effect of AahII toxin on slowing the fast inactivation of the Na_V_1.5 channel and allow to obtain values comparable to the control values (tau = 2 ± 0.6 ms), (Figures 2A,B; *n* = 5, ***p < 0.001). In contrast, at a lower ratio (1:0.5) ([AahII] = 300 nM: [Nb10] = 150 nM), the toxin–antibody complex reduced the toxin effect on inactivation slowdown (Figures 2A,B; *n* = 5, ***p < 0.001). It is also worth noting that neither Nb10 alone nor the AahII–Nb10 complexes affect the current amplitude (Figure 2C; *n* = 5, ***p < 0.001).

In order to investigate whether the perfusion of Nb10 is able to affect the binding of AahII on Na_V_1.5, we established a protocol to follow the inactivation time constant (tau) through different conditions (Figure 2D; *n* = 3, ***p < 0.001). After current stabilization by perfusion of the control solution (tau = 2.4 ± 0.4 ms), [AahII] = 75 nM was perfused throughout 7 min until the current stabilization (tau = 9.7 ± 1.2 ms); from 7 min up to 14 min, cells were perfused by a 1:1 AahII–Nb10 ratio at 75 nM (75 nM AahII +75 nM Nb10). The application of Nb10 promotes a partial abolishment of AahII action (tau = 4.7 ± 0.8 ms, Figures 2D–F; *n* = 3, ***p < 0.001).

Similar results are found when we perfused first 75 nM AahII during 7 min (tau = 9.6 ± 0.9 ms), followed by the perfusion of 75 nM Nb10 alone until 14 min (tau = 4.6 ± 0.4) (Supplemental Figures S2D–F, *n* = 3, *p* < 0.001).

These results strongly suggest that Nb10 can displace the AahII toxin from its binding site on the Na_V_1.5 channel.

### Nb10 Reverses the Effect of AahII on MDA-MB-231 Cell Invasion of Breast Cancer Cells

**FIGURE 3:**
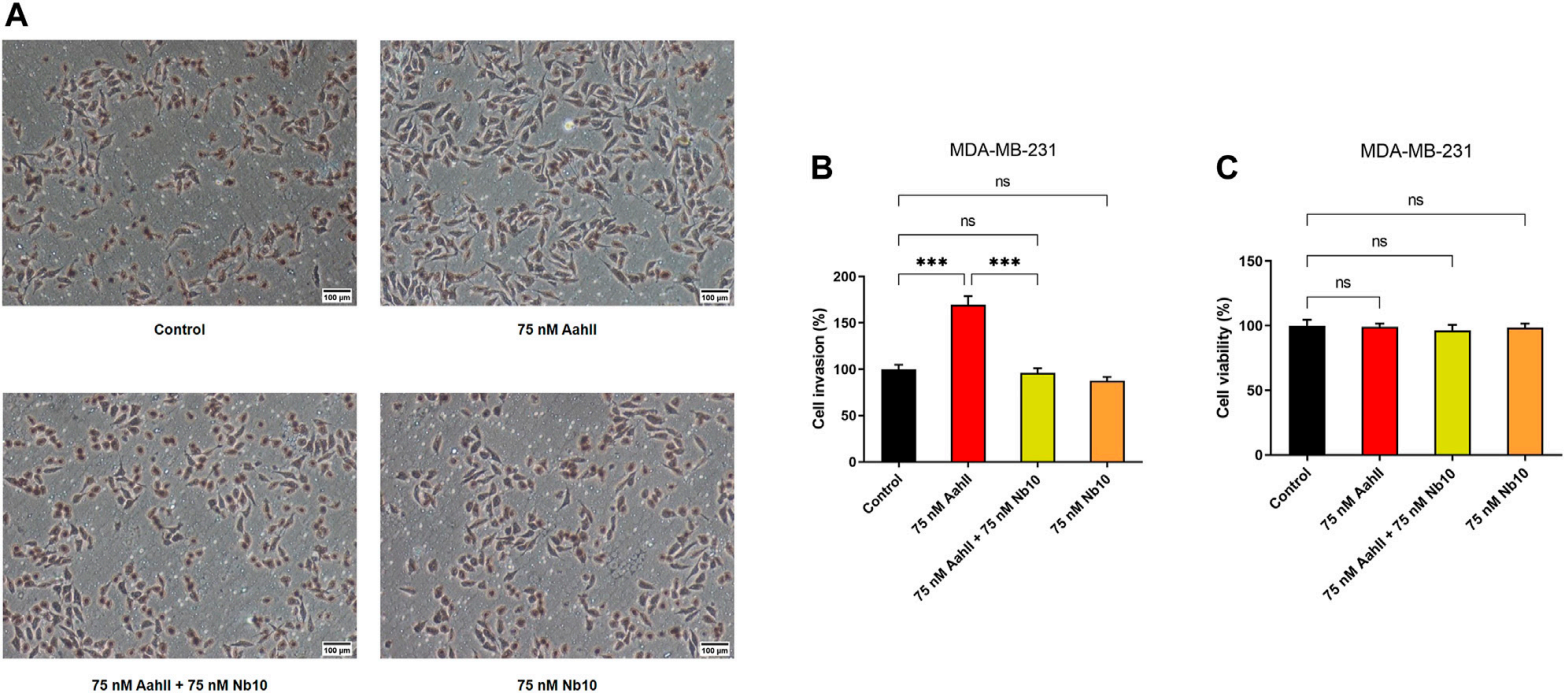
Nb10 can reverse the effect of AahII on MDA-MB-231 cell invasion. **(A)** Microscopic pictures of fields at different conditions. **(B)** Cell invasion was measured after 24 h of incubation in the control condition (control) and in the presence of AahII and Nb10 alone or combined at a 1:1 ratio. Results shown are representative of independent experiments, *n* = 3. All data are presented as mean ± SE (***p < 0.001). **(C)** Cell viability was performed in the same condition as **(B)**. Results shown are representative of independent experiments, *n* = 3. All data are presented as mean ± SE (*ns, p* > 0.05).

We investigated the effect of AahII and Nb10 alone and together on MDA-MB-231 cell invasion in the Boyden chamber. Nb10, alone, at 75 nM was without effect on MDA-MB-231 cell invasion (Figures 3A,B). At 75 nM, AahII alone increases cell invasion by 67.16 ± 9% (Figures 3A,B; *n* = 3, ***p < 0.001). In contrast, AahII failed to affect invasion in the presence of Nb10 at a 1:1 ratio (Figures 3A,B; *n* = 3, ***p < 0.001). Moreover, both AahII and Nb10 alone and the AahII:Nb10 ratio have no effect on proliferation (Figure 3C; *n* = 3, ***p < 0.001).

### Structural *in-Silico* Simulation of Na_V_1.5-AahII Toxin Interaction Complex

Molecular simulations were designed based on a benchmarking approach to predict the Na_V_1.5–AahII complex of interaction. Several molecular docking methods and classes were tested. Both rigid and flexible dockings were performed in blind and driven manners. In total, around 4,500 complexes were generated (data not shown). The best complex orientation was found with the flexible high ambiguity driven protein–protein docking (HADDOCK). The orientation has been established according to extracellular topological domains of the Na_V_1.5 ⍺-subunit. A total of 150 generated complexes were obtained and grouped into five main clusters. Cluster 1 is the largest with 121 among the 150 complexes containing the best-score docking-oriented complex with a HADDOCK score of -100.9. Data of generated complexes are shown in Table 1.

**TABLE 1:** Molecular docking (Na_V_1.5–AahII) HADDOCK grouped 150 structures into 5 clusters, representing 94.0% of the water-refined HADDOCK models. The statistics of the top 5 clusters are presented in this table. The top cluster is the most reliable according to the HADDOCK score. The Z-score indicates the number of standard deviations from the mean of this cluster in terms of score (the most negative HADDOCK score is the best).

Top 10	Cluster 1	Cluster 3	Cluster 2	Cluster 6	Cluster 4	Cluster 5
Nr 1 best complexes						
HADDOCK score	−100.9 ± 6.8	−79.0 ± 6.9	−72.7 ± 7.3	−70.7 ± 10.7	−64.0 ± 14.9	−58.5 ± 6.9
Cluster size	121	9	6	4	4	6
RMSD from the overall lowest-energy structure	1.4 ± 0.1	2.5 ± 0.1	2.9 ± 0.2	2.7 ± 0.1	2.2 ± 0.3	1; 8 ± 0.0
Van der Waals energy	−50.8 ± 5.8	−53.1 ± 3.6	−53.4 ± 8.2	−68.7 ± 1.0	−49.2 ± 5.5	−58.6 ± 4.9
Electrostatic energy	−84.6 ± 41.0	−10.6 ± 11.3	−9.1 ± 43.7	−9.0 ± 55.2	−8.1 ± 59.0	−6.4 ± 30.7
Desolvation energy	−34.8 ± 8.0	−28.7 ± 5.7	−25.2 ± 7.6	−10.8 ± 2.9	−24.1 ± 9.4	−17.7 ± 12.2
Restraint violation energy	16.3 ± 14.14	49.3 ± 47.15	59.3 ± 50.53	64.5 ± 33.83	69.9 ± 153.62	70.3 ± 98.97
Buried surface area	1538.6 ± 119.9	1737.9 ± 50.4	1760.9 ± 129.3	2147.0 ± 124.8	1753.7 ± 82.6	2072.7 ± 61.6
Z-score	−2.2	−0.6	−0.5	−0.3	0.1	0.3

**FIGURE 4:**
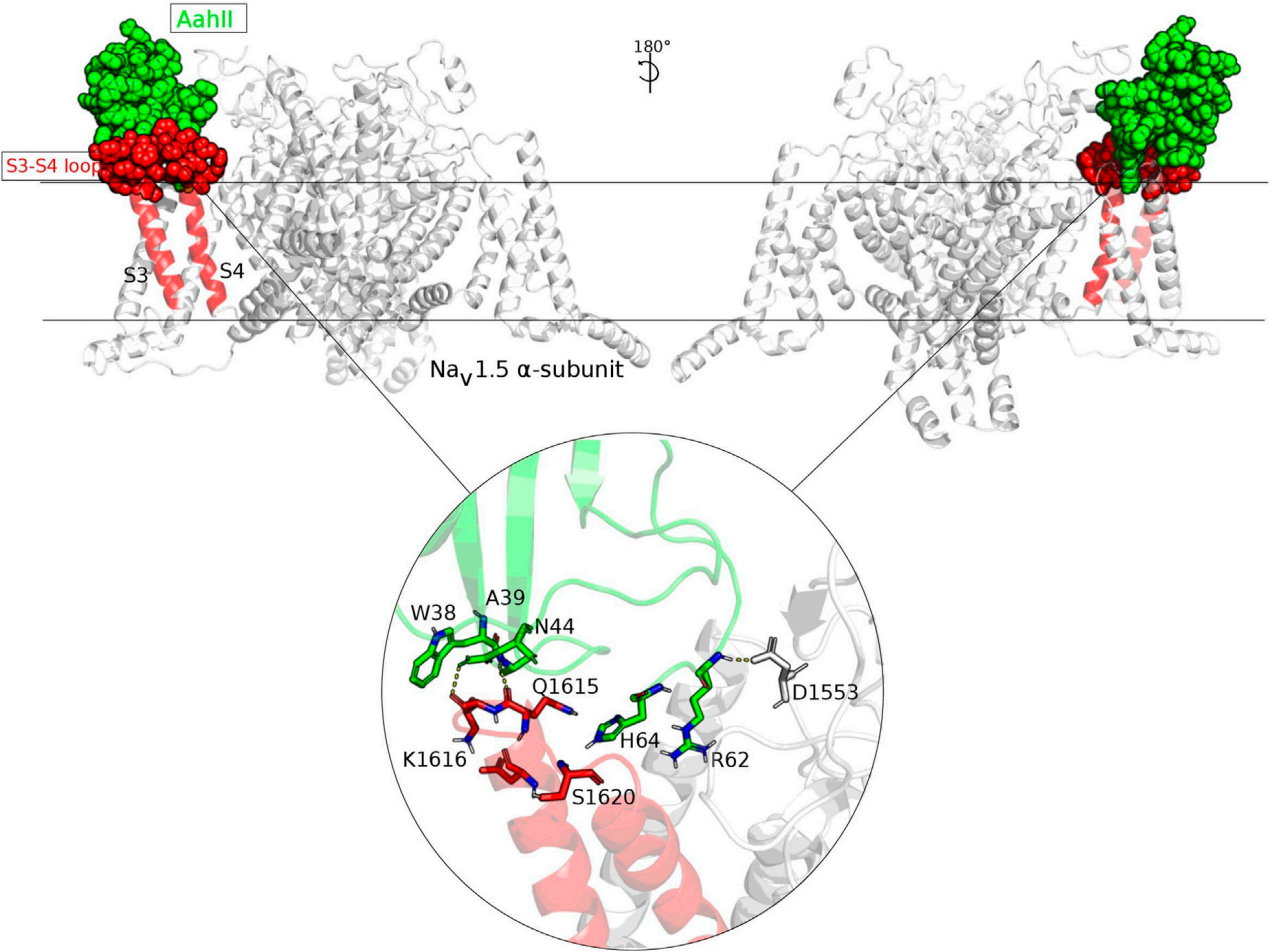
Two faces of the Na_V_1.5–AahII molecular interaction presentation. The two faces of the Na_V_1.5–AahII molecular interaction are presented with an orientation of 180°. The subunit involved in the interaction was colored in red and AahII scorpion toxin in green. The extracellular S3–S4 loop part of the subunit interacting with AahII is presented in spheres. This molecular orientation is selected among hundreds of poses according to a very accurate molecular docking approach. The zoomed part illustrated in a circle shows the most crucial residues involved in the complex interaction and maintaining a stable molecular orientation. These residues make H-bounds, as well as hydrophobic and hydrophilic interactions.

The best complex conformation shows the AahII scorpion toxin oriented to the S3–S4 extracellular loop (1,608–1,620). In general, this part of the Na_V_1.5 ⍺-subunit is dedicated to ⍺-toxins’ interactions. The selected stable orientation is based on the strong molecular interaction manner. First, the 3D complex showed that the Ala39 residue of AahII plays a crucial role to establish a stable bond through H-bound interactions with Na_V_1.5 ⍺-subunit Gln1615 and Lys1616 residue positions. A second significant interaction involves an H-bound interaction between the AahII Arg62 residue with the Asp1553 of the Na_V_1.5 ⍺-subunit extracellular topological domain (S1–S2 loops) (Figure 4).

## Discussion

The pharmacology of scorpion α-toxins is a great gateway to studying the structure and function of ion channels in order to increase our knowledge of their properties. In this report, we studied and characterized the α-scorpion toxin AahII mode of interaction with the Na_V_1.5 channel and the modulation of the complex interaction by Nb10, a highly neutralizing anti-AahII nanobody (Abderrazek et al., 2009). For this accomplishment, we used a functional Na_V_1.5 channel alpha subunit vector of an adult isoform transfected in HEK293.

Significantly, the AahII toxin slows the fast inactivation of the Na_V_1.5 channel by a factor of 4 at 75 nM while current amplitude was not affected, demonstrating its nanomolar ranging effect. Our results agree with a recently reported study showing that AahII toxin slows the Na_V_1.7 fast inactivation mechanism when used at 300 nM (EC_50_ = 51.7 ± 1.5 nM) (Clairfeuille et al., 2019). Their investigations of the cryo-EM-based structural AahII-Na_V_1.7 channel interaction showed that α-scorpion toxin AahII binds to two different sites on a nonfunctional chimera in both DI and DIV. However, they did not demonstrate whether AahII bound to either of these sites was functionally active in the chimera (Clairfeuille et al., 2019). In this paper and using a wash perfusion protocol to follow the kinetics of Na_V_ inactivation, we demonstrated that AahII slows the time inactivation of Na_V_1.5 at a low concentration (75 nM) and that the binding of AahII is extracellular.

In the past, pharmacological studies retained a single neurotoxin receptor (site 3) per Na_V_ channel located in VSD4 at which related gating modifier toxins, such as scorpion α-toxins and sea anemone toxins, bind (Rogers et al., 1996; Gordon et al., 2007). To address this question, we performed a Na_V_1.5 site 3 driven docking with AahII and generated 4,500 complexes from which the best orientation showed the involvement of the AahII C-terminal (Arg62 and His64) in the interaction with the DIV-S4 (1,575–1,615 residues) of Na_V_1.5. Our data are in accordance with a recent similar study that focuses on LqhIII α-toxin from the deathstalker scorpion *Leiurus quinquestriatus hebraeus* and the Na_V_1.5 channel, showing that LqhIII binds at the extracellular end of the aqueous cleft formed by S1–S2 and S3–S4 helical hairpins in VSD4 through its β2β3 loop and its C-terminal domain (Jiang et al., 2021). Other structure–activity relationship studies show that AahII interacts with Na_V_ channels through the β2–β3 loop from Gln37 to Pro60 (Clairfeuille et al., 2019) and its C-terminal segment especially involving Arg62 and His64 which are critical for potent modulation of Na_V_ channels by targeting neurotoxin receptor site 3 (Wang et al., 2003; Benkhadir et al., 2004; Clairfeuille et al., 2019). Indeed, the residues Phe15 and Trp38 and Asn44 from AahII establishing the β2–β3 loop have been described to recognize the VSD4 of the Na_V_ channels and play an important role in bioactivity of the α-toxin (Kharrat et al., 1989; Gur et al., 2011; Clairfeuille et al., 2019).

It is well admitted that scorpion α-toxins bind to the neurotoxin receptor in a voltage-dependent manner, with a high-affinity binding in the resting state (Clairfeuille et al., 2019; Jiang et al., 2021). Likely, depolarization of the channel reduces toxin affinity and causes its dissociation (Clairfeuille et al., 2019; Jiang et al., 2021). The binding of α-toxins prevents the outward movement of the gating charges of the DIV-S4 segment. In standard conditions, this normal outward movement of the DIV-S4 segment is followed by the unbending of the elbow formed by the DIV S4–S5 loop and the opening of the receptor site for binding of the IFM motif to guarantee the fast inactivation. The binding of AahII α-toxin would counteract this succession of conformational events that lead to fast inactivation.

We have previously demonstrated that Nbs are better tools to interact with toxins and neutralize their effects because of their small size, good stability, high level of expression in a prokaryotic systems, high solubility, and suitable specificity.

Following the development of a highly AahII-specific Nbs, we obtained Nb10 which was the “best-in-class” nanobody able to neutralize the most toxic scorpion α-toxin. This encouraged us to study and characterize the capacity of Nb10 to modulate the interaction of AahII with Na_V_1.5 using a whole-cell patch clamp. Nb10 was produced in a prokaryotic system and purified from *E. coli* bacterial periplasm that meets the required quality control standards (Abderrazek et al., 2009), which allowed us to have enough purified Nb10 quantity that specifically recognizes and binds AahII. Nb10 is a 14 kDa protein able to neutralize 7 LD_50_ AahII toxicity when injected in mice (association rate constant: K_on_ = 1.14 × 10^6^ M^−1^.S^−1^; dissociation rate constants: K_off_ = 5.69 × 10^–4^ s^−1^; equilibrium dissociation constant: K_D_ = 0.49 nM) (Abderrazek et al., 2009).

By an electrophysiological approach, we demonstrated that Nb10 can fully abolish the AahII effect on fast inactivation of the Na_V_1.5 channel, with a 1:1 ratio, while current amplitude was not affected. Indeed, perfusion kinetics monitoring of (AahII + Nb10) shows that Nb10 can abolish the AahII-binding action, strongly suggesting that Nb10 shifted or at least altered the AahII-binding site of interaction with VSD4, which releases the DIV-S4 segment to adopt its normal movement and initiates the rapid inactivation of the Na_V_1.5 channel.

Recent *in silico* 3D modeling of the interaction of AahII with Nb10 revealed the potential contribution of the Trp38 residue from AahII with Met103, Arg108, Tyr105, and Ala111 of Nb10 (Ksouri et al., 2018). Indeed, the segment from Ala39 to Ala45 in AahII has been identified as the main region responsible for antigenic reactivity (Devaux et al., 1993).

The *in-silico* prediction of the AahII–Nav1.5 alpha-subunit interaction showed that AahII binds at the extracellular end of the aqueous cleft formed by the S1–S2 and S3–S4 helical hairpins *via* its loop (from Trp38 to Asn44) and its C-terminal (Arg62 and His64). Our findings are comparable to the Cryo-EM experimentally solved results which revealed the structure of LqhIII bound to Na_V_1.5 at the 3.3 Å resolution, showing that the LqhIII scorpion toxin anchors on top of VSD4, wedged between the S1–S2 and S3–S4 linkers, and through the β2–β3 loop and the C-terminal of the LqhIII α-Scorpion toxins (Jiang et al., 2021). This confirms that α-Scorpion toxins bind to neurotoxin receptor site 3 in a voltage-dependent manner, with high-affinity binding to the resting state (Catterall 1977; Catterall 1979).

Despite the topological difference of the implicated amino acids in the interaction with the two distinct scorpion toxins (AahII and LqhIII), the positions of the bound toxin with the S1–S2 and S3–S4 helical hairpins are remarkably similar. Therefore, we can trust the structural outputs based on a very accurate computational approach. Herein, 3D modeling of the best AahII-Na_V_1.5 α-subunit complex showed that AahII Ala39 and Arg62 residues play a crucial role in establishing a stable interaction, through H-bounds, with Na_V_1.5 Gln1615 and Lys1616 residues.

These results indicated that Nb10 recognizes a similar or identical surface that directly interacts with the DIV-S4 segment of the Na_V_1.5 channel, involving two major residues at positions 39 and 62 in AahII. Indeed, the C-terminal AahII His64 and Arg62 are similarly involved in the Nb10–AahII and Na_V_1.5–AahII interaction complexes and participate in the AahII active site (Ksouri et al., 2018).

Furthermore, to address the need for a highly physiological characterization of the interaction between AahII and Nb10 on cells, we performed invasion assays using MDA-MB-231 cells that express a neonatal isoform of Na_V_1.5. This Na_V_1.5 channel is involved in both migration and invasion processes of the MDA-MB-231 triple-negative breast cancer cell line and promotes metastasis (Roger et al., 2003; Kamarulzaman et al., 2017; Zhang et al., 2018; Luo et al., 2020). Interestingly, as expected AahII increases the invasion of MDA-MB-231 cells by 67.16 ± 9%; the equimolar range of Nb10 can completely suppress this effect (1:1 M ratio). Therefore, both AahII and Nb10 may be useful for biomedical models assessing the involvement of Na_V_ channel subtypes in the invasion process capability of several tumor cell lines as well as drug delivery into tissues.

Altogether, our results suggest that Nb10 perfectly fits both AahII toxin-binding surfaces on the Na_V_ channel (β2–β3 loop and C-terminal domain) and dynamically blocks AahII–Na_V_1.5 interactions. The two amino acid positions in AahII are crucial for both interactions with Na_V_1.5 and Nb10.

## Materials and Methods

### Scorpion Venom Fractionation and AahII Toxin Purification

*Androctonus australis Hector* crude venom was extracted and purified by gel filtration chromatography according to the well-established protocol (Miranda et al., 1970) with slight optimization (Hmila et al., 2008) **(** Supplemental Figure S1A **)**. The toxic fraction containing toxins with molecular weight ranging from 3,000 to 7,500 Da named AahG50 was collected and then fractionated by FPLC (GE ÄKTA) on a cation exchange RESOURCE S chromatography column pre-equilibrated with of 0.05 M ammonium acetate buffer solution. Peptides were eluted over 80 min of linear gradient from 0.05 to 0.5 M ammonium acetate, pH 6.6, at a flow rate of 0.8 ml/min. Peptides are detected at an absorbance of 280 nm **(** Supplemental Figure S1B **)**. The toxic fraction 9 from FPLC was applied to a reverse-phase high-performance liquid chromatography (RT-HPLC) Dionex on a C8 column (Spherisorb 5 μm, L × I.D. 25 cm × 4.6 mm). The main peptides were eluted from the column at the rate of 0.8 ml/min using a three-step gradient. The initial mobile phase is 100% A [0.1% trifluoroacetic acid (TFA, Sigma-Aldrich, St. Louis, MO, USA) in water] and 0% B [0.1% TFA in acetonitrile (Sigma-Aldrich)]. The percentage of mobile phase B increases all over the three steps, from 0% to 20% over 4 min, beginning at 0 min; from 20% to 40% over 40 min, beginning at 4 min; and from 40% to 100% over 5 min, beginning at 44 min. Elution was performed at a flow rate of 0.8 ml/min. Absorbance was monitored at 280 nm **(** Supplemental Figure S1C **)**. The concentration of purified proteins was quantified by the QuantiPro BCA Assay Kit.

### Nb10 Expression and Purification

The recombinant vector displaying the VHH gene transcript encoding Nb10, subcloned into the pHEN6 expression vector using the restriction enzymes NcoI or PstI and BstEII, was used to transform non-suppressive and competitive *WK6 E. coli* electrocompetent cells that met the required quality control standards. The expression of Nb10 was performed as previously described (Abderrazek et al., 2009). The obtained periplasmic extract containing His-tagged proteins was incubated with nickel-Sepharose beads (Sigma-Aldrich) for 1 h at 4°C under gentle agitation. The mixture was then poured into a PD-10 column (Cytiva, Marlborough, MA, USA) and allowed to drain by gravity. The HIS-Select adsorbent was washed with 20 column volumes of PBS 1× and allowed to drain by gravity. The washing step ends when the OD280 nm of the last droplet of the eluate approaches 0. Elution of the Ni-ion-bound protein fraction was done by adding 5 ml of concentrated PBS/imidazole at 500 mM. The purity of the eluted protein was checked by SDS-polyacrylamide gel electrophoresis and visualized using Coomassie brilliant blue. The protein of interest has a molecular weight of about 14 kDa **(** Supplemental Figure 1D **)**. The final yield was determined from the UV absorption at 280 nm and the theoretical extinction coefficient of Nb10.

### Cell Cultures

HEK293 and MDA-MB-231 cells were cultured in Eagle’s Minimal Essential Medium (EMEM; Life Technologies, Saint Aubin, France) supplemented with 20 mM HEPES, 2 mM Glutamine (Life Technologies, Saint Aubin, France), and 5% fetal bovine serum (FBS, Life Technologies, Saint Aubin, France). The medium is changed every 48 h. Cells are kept in the incubator at 5% CO_2_ and 37°C and weekly trypsinized using trypsin-EDTA. The absence of *mycoplasma* is checked twice a month.

### Patch Clamp Experiments

HEK293 cells stably expressing Na_V_1.5 were generously gifted by Prof. Hugues Abriel (Institute of Biochemistry and Molecular Medicine, University of Bern, Switzerland). Cells were cultured in 35-mm Petri dishes at a density of 1 × 10^4^ cells at least 2 days before patch-clamp experiments. Before recording, cells were washed with the saline solution used as an extracellular medium for control conditions. Whole-cell patch-clamp recordings were obtained using a patch-clamp amplifier (Axon Axopatch 200B, Molecular Devices, Sunnyvale, CA, USA). The recording pipette intracellular solution contained the following (in mM): 8 Na-gluconate, 145 Cs-methane sulfonate, 10 EGTA, and 10 HEPES (pH 7.2 adjusted with NaOH, osmolarity 326 mOsm). The extracellular recording solution contained the following (in mM): 150 Na-gluconate, 5 K-gluconate, 2 Mg-gluconate, 2 Ca-gluconate, 10 HEPES, and 5 glucose (pH 7.4 adjusted with NaOH, osmolarity 337 mOsm). All electrophysiological experiments were performed at room temperature. The whole-cell recording of the patch-clamp technique was used with no coated pipettes (Hirschmann®, Laborgerate, Eberstadt, Germany) with resistance between 3 and 5 MΩ. Seal resistance was typically more than 1 GΩ before the break of the membrane to access the whole-cell configuration. Perfusion of different concentrations of AahII and Nb10 was performed using a perfusion system with a flow rate of 5 ml/min. Currents were recorded at the frequency of 0.2 Hz. Signals were filtered at 1 kHz and digitized at 5 kHz. Cells were voltage-clamped at −140 mV, and Na^+^ currents were recorded following membrane depolarization from −140 to −40 mV for 200 ms every 5 s. Cell capacitances were not compensated. Data were analyzed using Clampfit 10.7 (Molecular Devices, Inc., San Jose, CA, USA), then plotted in OriginPro 2018 (Software, Inc., USA).

The inactivation time constant (tau) value is calculated directly using a single exponential function fitting on Clampfit 10.7 (Molecular Devices, Inc., San Jose, CA, United States)

### Cell Invasion Assays

Cell invasion experiments were performed by Boyden chamber with Matrigel assay. The upper compartment that contains 0% FBS-treated medium was seeded with 4 × 10^4^ cells. The lower compartment contains a 5% FBS-treated medium to promote chemotaxis. After 24 h of incubation at 37°C, inserts were washed with PBS (Sigma-Aldrich, Darmstadt Germany), fixed 10 min by methanol (Sigma-Aldrich, Darmstadt Germany), and colored 5 min with hematoxylin (Sigma-Aldrich, Darmstadt Germany). Two supplementary washes with water are performed to eliminate the rest of hematoxylin. The remaining cells on the upper side were removed from the membrane by rubbing. Invasive cells in the lower compartment are then counted on 20 fields at ×20 magnification with an inverted microscope (Zeiss, Cambridge, England). For each experiment, the number of invasive cells per area for each condition was normalized by the mean of invasive cells in the control condition. Experiments are done in duplicate for each condition.

### Cell Viability Assays

Cell viability was performed by MTT assay as previously described (Lefebvre et al., 2020). Cells were seeded in 6-well plates in double of density as invasion assays at the rate of 8 × 10^4^ cells per well. After 24 h of incubation, the medium is replaced by 800 µl of MTT (tetrazolium salts of 3-(4,5-dimethyl-2-thiazolyl)-2,5-diphenyl-2H-tetrazolium bromide, Sigma-Aldrich, Darmstadt Germany) solubilized in culture medium without FBS at 0.5 mg/ml in each well. Plates were then incubated for 50 min at 37°C in obscurity. To dissolve purple formazan crystals formed by living cells, the culture medium was replaced with DMSO (dimethyl sulfoxide, Sigma-Aldrich, Darmstadt Germany). Absorbance of each well was quantified at 550 nm using an Infinite® 200 Pro Reader (Tecan Trading AG, Männedorf, Switzerland).

### Statistical Data Analysis

Data are presented as mean ± SEM, and *n* refers to the number of individual cells. All the experiments were performed in at least 3 different cell passages. Data analysis and figure conception were made using GraphPad Prism 9.0 (GraphPad Software, Inc., La Jolla, CA, USA), Origin 2018 (Microcal Software, Inc., Los Angeles, CA, USA), and Clampfit 10.7 (Molecular Devices, Inc., USA). The mean values of more than two groups were tested using a two-way analysis of variance (ANOVA) followed by Holm–Sidak *post hoc* tests. Differences between the values were considered significant when *p* < 0.05. The *p*-values < 0.05, <0.01, and <0.001 are represented as *, **, and ***, respectively.

### Structural *in Silico* Simulation of Na_V_1.5-AahII Interaction

The 3D structure of Na_V_1.5 at 3.2–3.5 Å resolution was extracted from the protein data bank (PDB ID: 6UZ3). The corresponding Na_V_1.5 channel structure enables at a functional level to generate cardiac action potentials and initiates the heartbeat. The Na_V_1.5 subunit α is described from 1 to 1,838 in the linear amino acid sequence. This structure was extracted, cleaned, then relaxed using several parsing PDB files, bioinformatics tools, and python scripts. The 3D crystal structure of toxin II from the scorpion *Androctonus australis Hector* refined at 1.3 Å resolution was extracted from the protein data bank (PDB ID: 1PTX). The flexible computational docking simulation was performed between AahII as a blinded driven molecule and Na_V_1.5 structures *via* its α-toxin-specific binding region of the DIV voltage sensor. The HADDOCK (high ambiguity driven protein–protein docking) was used as the driven flexible docking tool for modeling biomolecular complexes.

Restraint data to drive the docking as active residues were implicated without excluding surrounding surface residues or passive residues around the active residues, to avoid bias molecular interaction output information.

Linux command lines were used to generate molecular complexes. Clustering of molecular complexes based on a specific HADDOCK score allows the best understanding of interaction molecular behaviors; therefore, the best-oriented complex can be well selected based on several molecular docking features as the main component of the HADDOCK score, such as interface-RMSD calculated on the backbone (I-RMSD), ligand-RMSD calculated on the backbone atoms (l-RMSD), and fraction of common contacts (FFC).

The selected molecular complex was analyzed, and residues with crucial interactions were highlighted through an amino acid sequence contact map with a threshold of 5 Å.

Generated complex structures and interactions were then visualized *via* the molecular visualization software PyMOL (Schrödinger et al., 2020).

## Data Availability

The original contributions presented in the study are included in the article/Supplementary Material; further inquiries can be directed to the corresponding authors.
