# Supplementary material for: The Pharmacological and Structural Basis of the AahII–NaV1.5 Interaction and Modulation by the Anti-AahII Nb10 Nanobody
Source: Front Pharmacol. 2022 Feb 28;13:821181. doi: 10.3389/fphar.2022.821181 (PMC8918821; doi:10.3389/fphar.2022.821181)
Supplement: Supplementary file 1 [file DataSheet1.docx]

**SUPPLEMENTAL FIGURES**


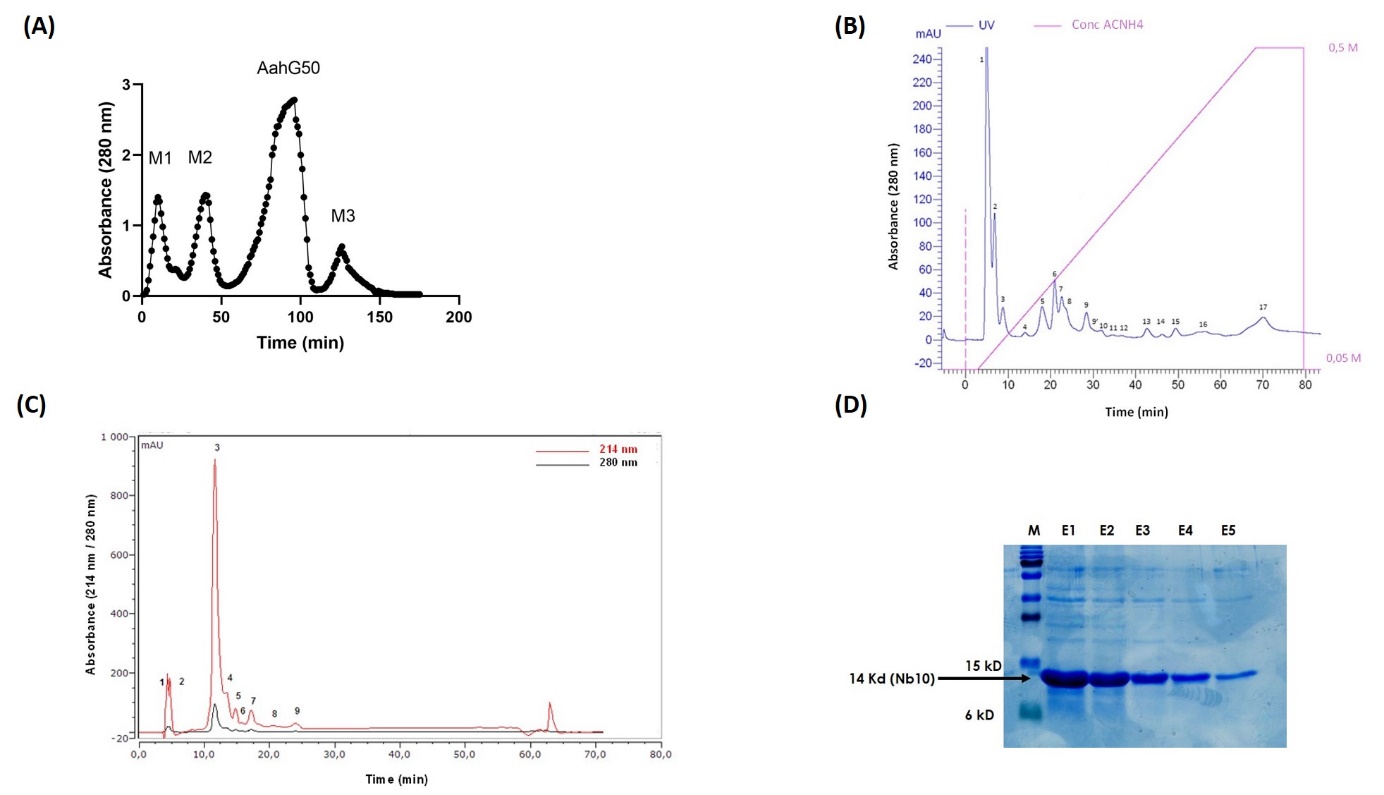


**Supplemental Figure S1. (A)** Crude venom was extracted with cold water (1:4 v/v), and centrifuged at 15 000xg for 15 min. The supernatant was loaded on Sephadex G-50 gel filtration chromatography column (K26/100) equilibrated with 0.1 M acetic acid, to obtain mainly 4 fractions. **(B)** The toxic fraction of the venom AahG50 was submitted to FPLC on a Mono S column pre-equilibrated with 0.05 M ammonium acetate buffer. Proteins were eluted with a 70 min linear gradient from 0.05 to 0.5 M ammonium acetate (pH 6.6) at a flow rate of 0.8 ml/min; absorbance was monitored at 280 nm. **(C)** The fraction 9 from FPLC was purified by RT-HPLC on a C8 column, as described in the experimental section, AahII toxin was eluted at 12 min of retention time (Peak 3). **(D)** SDS-Page check of Nb10 (MW 14 kDa) production and purification following Elutions (E).


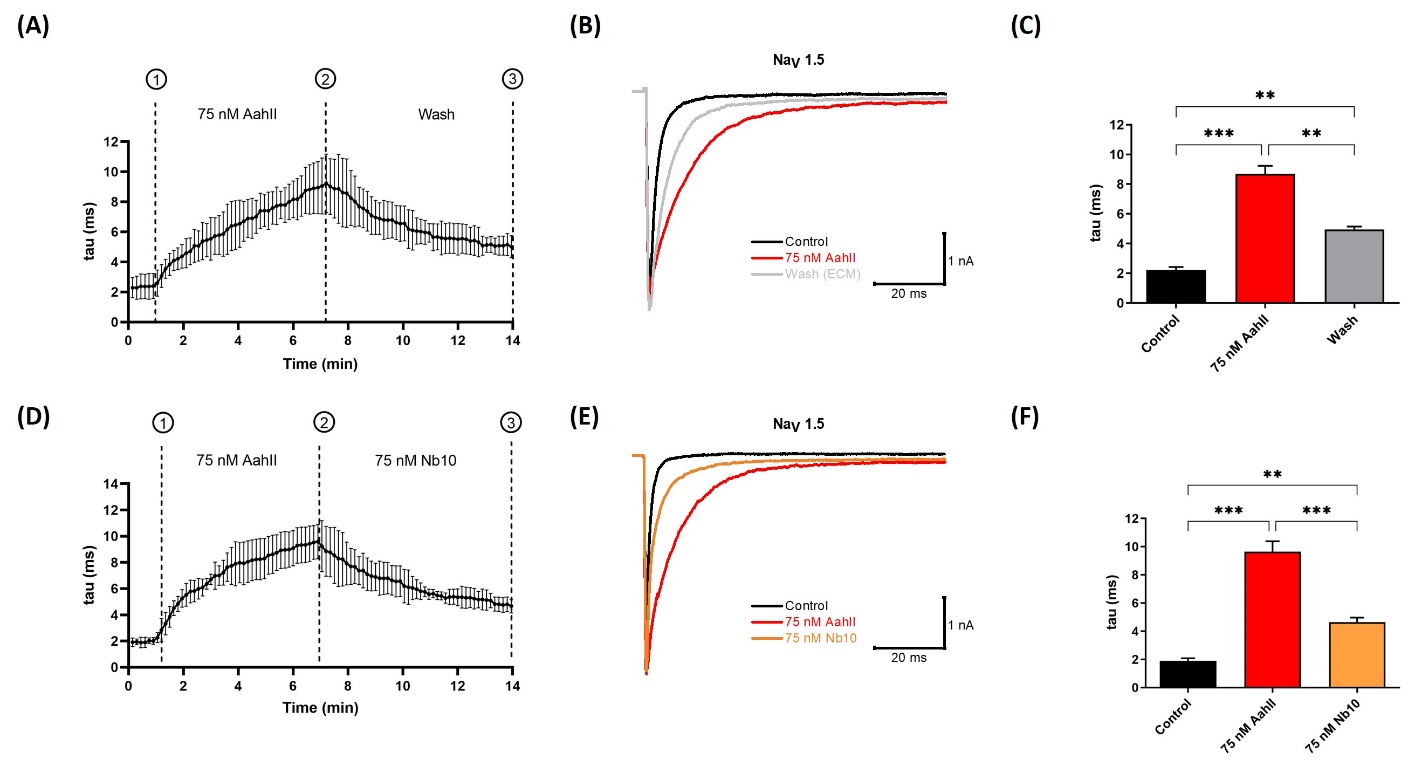


**Supplemental Figure S2. (A)** Mean of kinetics of inactivation time constant of Na_V_1.5 channel (tau) following perfusion of AahII toxin at 75 nM from 1 to 7 min followed by washing with ECM up to 14 min. Traces are representative of independent experiments, *n* = 3. All datas are presented as mean ± standard error. **(B)** Representative of original whole-cell current traces showing the reversible effect of AahII observed in panel E. Black trace : Current trace recorded in control medium, Red trace : Current obtained at 7 min represents the maximum effect of AahII toxin at 75 nM, grey trace : Current trace after washing the AahII toxin up to 14 min. Traces are representative of independent experiments, *n* = 3. **(C)** Mean of inactivation time constant of Na_V_1.5 channel (tau) following a similar protocol as in panel (A).

**(D)** Mean of kinetics of inactivation time constant of Na_V_1.5 channel (tau) following perfusion of AahII toxin at 75 nM from 1 to 7 min followed by perfusing of 75 nM Nb10 up to 14 min. Traces are representative of independent experiments, *n* = 3. All datas are presented as mean ± standard error **(E)** Representative of original whole-cell current traces showing the reversible effect of AahII observed in panel E. Black trace : Current trace recorded in control medium, Red trace : Current obtained at 7 min represents the maximum effect of AahII toxin at 75 nM, orange trace : Current trace after perfusing the 75 nM Nb10 up to 14 min. Traces are representative of independent experiments, *n* = 3. **(F)** Mean of inactivation time constant of Na_V_1.5 channel (tau) following a similar protocol as in panel (D).
